# FEEL YOUNGER, THINK YOUNGER? THE RELATIONSHIP BETWEEN MOMENTARY FLUCTUATIONS OF SUBJECTIVE AGE AND MIND-WANDERING

**DOI:** 10.1093/geroni/igz038.201

**Published:** 2019-11-08

**Authors:** Matthew L Hughes, Dayna Touron

**Affiliations:** The University of North Carolina at Greensboro, Greensboro, North Carolina, United States

## Abstract

Subjective age is susceptible to day-to-day influence from external factors (e.g., stress), but it is not known how it fluctuates throughout the day. Mind-wandering, when one’s concentration is not on the task at hand, can be induced in a laboratory, but less is known about older adults’ mind-wandering in everyday life. This pre-registered study used experience sampling to investigate the relationship between mind-wandering and subjective age. Participants ages 50 years and older carried iPods for 7 days. Participants received 8 daily probes asking questions about mind-wandering and subjective age. Subjective age was assessed using an unmarked sliding scale between 0 and 120 years. For the first time, we demonstrated subjective age fluctuation within each day, with an average coefficient of variation of 12.40 (range 9.73–47.56). Participants also reported mind wandering about 34% of the time. Multilevel modeling provides insight into the relationship between everyday fluctuations of subjective age and mind-wandering.

